# Longitudinal Follow-Up of Children Born Preterm: Neurodevelopment From 2 to 10 Years of Age

**DOI:** 10.3389/fped.2021.674221

**Published:** 2021-06-21

**Authors:** Lisette Jansen, Cacha M. P. C. D. Peeters-Scholte, Annette A. van den Berg-Huysmans, Jeanine M. M. van Klink, Monique Rijken, Janneke C. van Egmond-van Dam, Robert R. J. M. Vermeiren, Sylke J. Steggerda

**Affiliations:** ^1^Department of Medical Psychology, Leiden University Medical Center, Leiden, Netherlands; ^2^Department of Child and Adolescent Psychiatry, Leiden University Medical Center, Curium, Leiden, Netherlands; ^3^Department of Neurology, Leiden University Medical Center, Leiden, Netherlands; ^4^Department of Radiology, Leiden University Medical Center, Leiden, Netherlands; ^5^Department of Neonatology, Leiden University Medical Center, Leiden, Netherlands; ^6^Department of Physical Therapy, Leiden University Medical Center, Leiden, Netherlands

**Keywords:** preterm birth, long-term outcome, long term follow-up, longitudinal, premature birth, developmental domains

## Abstract

**Objective:** To investigate the rate and stability of impairments in children born preterm by assessing (1) early and school-age outcome in four developmental domains and (2) individual changes in outcome at both timepoints.

**Design:** Prospective, longitudinal cohort study in children born in 2006–2007, <32 weeks' gestation. Follow-up at 2 and 10 years of age included standardized neurological, motor, cognitive and behavioral assessments. Children were categorized as having no, mild or moderate-severe impairment in these four domains. A composite impairment score was composed and the number of domains with impairments counted. For each child, individual outcomes at both timepoints were compared.

**Results:** Follow-up at both time-points was available in 71/113(63%) children. At group level, there were no significant changes in the severity of impairments per domain. However, at individual level, there were less children with a mild abnormal composite score at 10 years of age (44 vs. 20%; *p* = 0.006), and more with a moderate-severe abnormal composite score (12 vs. 35%; p = 0.001). Especially children with normal/mild outcome at 2 years were likely to shift to other outcome categories over time.

**Conclusions:** Children with early severe impairment are likely experiencing impairments later on, but early normal/mild abnormal outcomes should be interpreted with care, considering the large individual shifts over time. Long-term follow-up in all children born very preterm should therefore be continued to at least school-age.

## Introduction

Being born prematurely threatens a healthy development across the life course (1). With developmental challenges arising, or becoming more visible with increasing age and additional demands on the child's functioning, favorable outcomes at an early age do not necessarily reflect on a child's abilities later in life (2). In order to fully recognize the difficulties of children born preterm, it is important to follow them individually, through several developmental stages, and include a standardized assessment of multiple domains of functioning.

While studies initially reported on major handicaps, including sensory deficits, cerebral palsy and cognitive delay, the focus has shifted over the past years to a broader range of milder impairments. Alongside, the assessment of impairment across multiple domains has received more attention and led to the understanding that children born preterm are prone to develop impairments in several domains of functioning at the same time (3). This combination of multiple (mild or severe) impairments in different domains might have a significant impact on functioning later on in life.

Several follow-up studies have now reported on outcomes in longitudinal cohorts of children born preterm at two or more time-points, however the majority of them only reported outcomes at group level, discarding the possible existing variation at an individual level (2, 4–7). Studies that did report on individual developmental trajectories mainly focused on one specific outcome domain in cohorts of children born extremely preterm (<26 weeks gestation) and reported stable trajectories of unfavorable cognitive and behavioral outcomes, persisting into adulthood (8, 9). Prospective studies using standardized outcome assessments of individual trajectories over time in multiple developmental domains are limited. One study reported stable numbers of children with severe disabilities (cerebral palsy, moderate to severe impairment in neuromotor function, vision, hearing and/or cognition) between 6 and 11 years of age, with considerable variation in individual trajectories (10). Recently, a study by Taylor and colleagues (11) reported a weak relationship between neurodevelopmental impairment at 2 and 10years of age in a large cohort of infants born extremely preterm (<28 weeks gestation). In this study, a relatively high proportion of children had an improvement of their neurodevelopmental impairment classification between infancy and childhood. Combining knowledge on the outcomes at different time-points and the individual variation in multiple domains between 2 and 10 years of age is important, not only for children born extremely preterm, but also for those born very preterm (28–32 weeks gestation). On one hand, it will benefit the counseling of parents of children born premature by potentially identifying those at risk, and, on the other hand, it may support early intervention strategies to improve outcome.

The aim of the current study was to investigate the rate and stability of impairment in a prospective cohort of children born preterm (<32 weeks' gestation) between May 2006 and November 2007, by standardized assessment of neurological, motor, cognitive, and behavioral outcomes at both 2 and 10 years of age. A second aim was to assess the composite impairment scores and to report on the co-occurrence of impairments by considering the number of affected domains for each child at both timepoints. The final aim was to assess the individual changes in outcome in the separate domains over time. This will give an insight in the rate and stability of impairments in preterm born children from toddler to school-age.

## Methods

### Participants

This study was performed as part of a larger single-center longitudinal study on neuroimaging and outcome after preterm birth. For this study, a cohort of 113 children born preterm (<32 weeks' gestation), who were admitted to the tertiary neonatal unit of Leiden University Medical Center (LUMC) between May 2006 and November 2007, was included. Neonatal in- and exclusion criteria of the original cohort were published previously (12, 13). For this particular follow-up study, only the children with follow-up data at both 2 and 10 years of age were included. Children who were unable to be assessed due to severe motor, visual or cognitive disabilities were assigned the corresponding lowest score.

### Outcome Assessment

Children and their parents were invited for follow-up visits at 2 years of age corrected for prematurity and at 10 years of age. Both visits consisted of a standardized pediatric, neurologic, cognitive and motor functioning examination. Parents reported on the presence of problem behavior. All tests were performed by certified professionals, as part of a regular, standardized clinical follow-up program, according to the national guideline of the Dutch working group on follow-up for preterm infants. The assessors at 10 years of age had no knowledge of outcomes at 2 years of age prior to their assessment. Outcomes of this cohort at 2 years of age, in relation to brain imaging findings, have been published previously (12, 14–16). The institutional review board of the LUMC approved this study, and written parental consent was obtained from both parents (P06.002). For the follow-up at 10 years of age, a waiver was obtained as this is part of the national clinical follow-up program (C15.072/P17.087).

### Baseline Characteristics of the Study Population

Perinatal data were available for all children, as published earlier (13). Small for Gestational Age (SGA) was based on birth weight <10^th^ percentile (17). Postnatal sepsis was determined by a positive blood culture. Necrotizing Enterocolitis (NEC) was diagnosed when stage ≥2 was present (18). Bronchopulmonary Dysplasia (BPD) was categorized as either none or mild/moderate/severe BPD (19). Intraventricular Hemorrhage (IVH) severity was based on neonatal cranial ultrasound as low grade (grade I–II) or high grade (grade III and/or periventricular hemorrhagic infarction) (20). White matter and cerebellar injury were classified according to a standardized MR imaging scoring system (21). Maternal education was classified as low (primary school and lower general secondary school), intermediate, or high (higher vocational school and university) (22).

### Measures

The children were seen by a certified neonatologist, pediatric physical therapist, and child psychologist at 2 and 10 years of age, and, at 10 years of age, also by a child neurologist. Neurological functioning at 2 years of age was assessed by a standardized neurological examination according to Hempel which assesses the following clusters: fine and gross motor functioning, posture and muscle tone, reflexes, and visuomotor functioning (23, 24). At 10 years of age, neurological functioning was examined according to Touwen which assesses the following clusters: involuntary and associated movements, posture, reflexes, sensory deficits and cranial nerve dysfunction (25). Children were classified as having simple minor neurological dysfunction (MND) with one dysfunctional cluster, or complex MND with two or more dysfunctional clusters.

The presence and grade of CP at 2 and 10 years was examined using the Gross Motor Function Classification Score (GMFCS) (26). A GMFCS score of ≥2 was considered mild to severe CP.

Motor development at 2 years of age was examined using the fine and gross motor scale of the Bayley Scales of Infant and Toddler Development, 3^rd^ edition (Bayley-III) (27, 28). At the time of assessment, American norms were used due to the lack of a Dutch norm group. Using the American norms leads to an underestimation of developmental delays at 2 years of age (29, 30), and therefore the Bayley-III motor scores were corrected for the current Dutch norms (31). At 10 years of age, the Movement Assessment Battery for Children, 2^nd^ Edition (M-ABC-II) (32) was administered and assessed according to Dutch norms. According to the Bayley-III, children had either a mild developmental delay with a motor score between 1 and 2 standard deviation (SD), or a moderate-severe developmental delay with a motor score of >2 SD below the mean. According to the M-ABC-II, children were classified mildly abnormal with scores between the 5^th^ and 15^th^ percentile, or moderate-severe abnormal with scores ≤ 5^th^ percentile.

Cognitive ability at 2 years of age was assessed using the Bayley-III cognitive scale (27). Similar to the Bayley-III motor score, the cognition scores were originally derived from American norms, and therefore corrected for the current Dutch norms to avoid underestimation. At 10 years of age, the Wechsler Intelligence Scale for Children (WISC-III) was administered (33). Children were classified as mildly abnormal with a score between 1 and 2 SD, or moderate-severe abnormal with a score of > 2 SD below the mean.

Parents reported on behavioral problems at both timepoints by means of the Child Behavioral CheckList (CBCL) (34). Age standardized t-scores were obtained for internalizing, externalizing and total problem behavior, where higher scores indicate higher levels of problem behavior. Children were classified with mild behavioral problems, with t-scores in the borderline clinical range (≥84^th^ percentile), or moderate-severe behavioral problems with t-scores in the clinical range (≥90^th^ percentile).

### Composite Impairment Score

A composite impairment score for both timepoints was created. Children were categorized into three groups: no impairment, mild impairment or moderate-severe impairment. Mild impairment was defined as having at least one mild impairment in neurological, motor, cognitive or behavioral functioning, in absence of any moderate-severe impairment. Moderate-severe impairment was defined as having at least one moderate-severe impairment in one of the above mentioned domains. The composite impairment score included children with one, as well as those with more than one abnormal domain.

### Multidomain Impairment Scores

For the multidomain impairment score, the number of domains in which a child experienced impairments was counted, ranging from zero (no impairments at all) to four (impaired in the neurological, motor, cognitive and behavioral domain). Multidomain impairment was present when a child had a mild or moderate-severe outcome in two or more domains.

### Developmental Change

In order to assess the developmental change, we investigated differences over time on both a group level and an individual level for each domain separately. Children were categorized as normal, mild, or moderate-severe at both timepoints for each domain, making it possible to investigate categorical shifts for each separate domain over time.

### Statistical Analysis

Statistical analyses were conducted using SPSS version 23.0 (IBM, Armonk, NY, USA). Descriptive results for nominal variables were presented as number of cases and percentages. Means and SD's were reported for continuous variables. Perinatal factors of preterm children with and without follow-up were compared to assess if selective loss to follow-up occurred. To assess whether there was a difference in group distribution of the composite impairment score and multidomain impairment score, and to adjust for the effect of paired testing, the marginal homogeneity test was conducted. If so, McNemar tests were conducted *post-hoc*. A Bonferroni correction was conducted to adjust for multiple comparisons, leading to a significant *p*-value of < 0.013 (0.05/4). The number of domains between 2 and 10 years of age was compared with a paired *t*-test. The individual changes within the different domains were assessed with a Chi Square test.

## Results

The original cohort consisted of 113 children. Follow-up assessment at 2 years of age was available for 86 children (76%), of whom 71 children were also assessed at 10 years of age. Baseline characteristics of the 71 participating children are shown in Table 1. There were no differences in clinical parameters between children with and without follow-up at both timepoints (no follow-up or available only at one timepoint, *n* = 42). Due to severe motor and cognitive disability, four children were unable to complete the neurological, motor, and cognitive follow-up assessment at 10 years of age, and were therefore assigned the lowest score (complex MND within the neurological domain and ≥2 SD below the mean within the motor and cognitive domain). Their parents did complete the behavioral questionnaire. Table 2 shows the outcomes of the neurological, motor, cognitive and behavioral assessments at both time points.

**Table 1:** Perinatal characteristics and level of maternal education of the study population.

**Perinatal characteristics**	**Participants (*n* = 71)**	**No follow-up available at both timepoints (*n* = 42)**	***P***
Male sex (%)	38 (54%)	29 (69%)	0.106
Part of twins or triplets (%)	23 (32%)	13 (31%)	0.117
GA (weeks), mean ± SD	29.2 ± 2.0	28.7 ± 2.0	0.226
BW (g), mean ± SD	1234 ± 365	1178 ± 358	0.435
SGA (%)	8 (11%)	4 (10%)	0.585
**BPD (%)**			
Mild Moderate/severe	13 (18%) 16 (23%)	11 (26%) 11 (26%)	0.250
Sepsis (%)	27 (38%)	17 (41%)	0.698
NEC (%)	1 (1%)	2 (5%)	0.342
Low grade IVH	12 (17%)	6 (14%)	0.636
High grade IVH	7 (10%)	4 (10%)	0.954
**White matter injury**			
Mild Moderate/severe	14 (20%) 18 (26%)	15 (36%) 9 (22%)	0.965
**Cerebellar injury**			
Mild Moderate/severe	9 (13%) 6 (9%)	3 (7%) 6 (14%)	0.183
**Maternal education**		*n* = 29^*^	
Low (%)	18 (25%)	5 (18%)	0.860
Intermediate (%)	22 (31%)	13 (45%)	
High (%)	31 (44%)	11 (38%)	

* *Information was available for 29 children*.

*SD, standard deviation; GA, gestational age; BW, birth weight; SGA, small for gestational age based on a birth weight below the 10th percentile; BPD, bronchopulmonary dysplasia; postnatal sepsis, with a positive blood culture; NEC, necrotizing enterocolitis ≥ stage 2; IVH, intraventricular hemorrhage*.

**Table 2:** Outcomes at 2 and 10 years of age.

	**2 years of age**	**10 years of age**	***P***
Neurological examination	Hempel *(n = 71)*	Touwen (*n* = 70)	0.662
Normal	45 (63%)	46 (66%)	
Mild MND	19 (27%)	13 (18%)	
Complex MND	7 (10%)	11 (16%)	
Motor outcome	BSID (*n* = 65)	M-ABC (*n* = 69)	0.201
Mean ± SD	98.9 ± 15.5		
Standard Score ± SD		9.5 ± 3.2	
Normal	53 (81%)	56 (82%)	
Mild impairment	7 (11%)	1 (1%)	
Moderate-severe impairment	5 (8%)	12 (17%)	
Cognition	BSID-III (*n* = 70)	WISC-III (*n* = 71)	0.072
Mean ± SD	98.4 ± 16.6	95.3 ± 17.0	
Normal	56 (81%)	51 (72%)	
Mild impairment	10 (15%)	15 (21%)	
Moderate-severe impairment	3 (4%)	5 (7%)	
Behavior total	CBCL (*n* = 66)	CBCL (*n* = 65)	0.182
Mean ± SD	50.1 ± 8.3	51.0 ± 11.2	
Normal	57 (86%)	51 (78%)	
Mild impairment	6 (9%)	7 (11%)	
Moderate-severe impairment	3 (5%)	7 (11%)	
CP	(*n* = 71) 5 (7%)	(*n* = 71) 5 (7%)	1.000

*BSID, Bayley's scale of infant and toddler development; CBCL, child behavior checklist; CP, cerebral palsy; M-ABC, movement assessment battery for children; WISC, Wechsler intelligence scale for children; SD, standard deviation*.

### Composite Impairment Score

**Figure 1:**
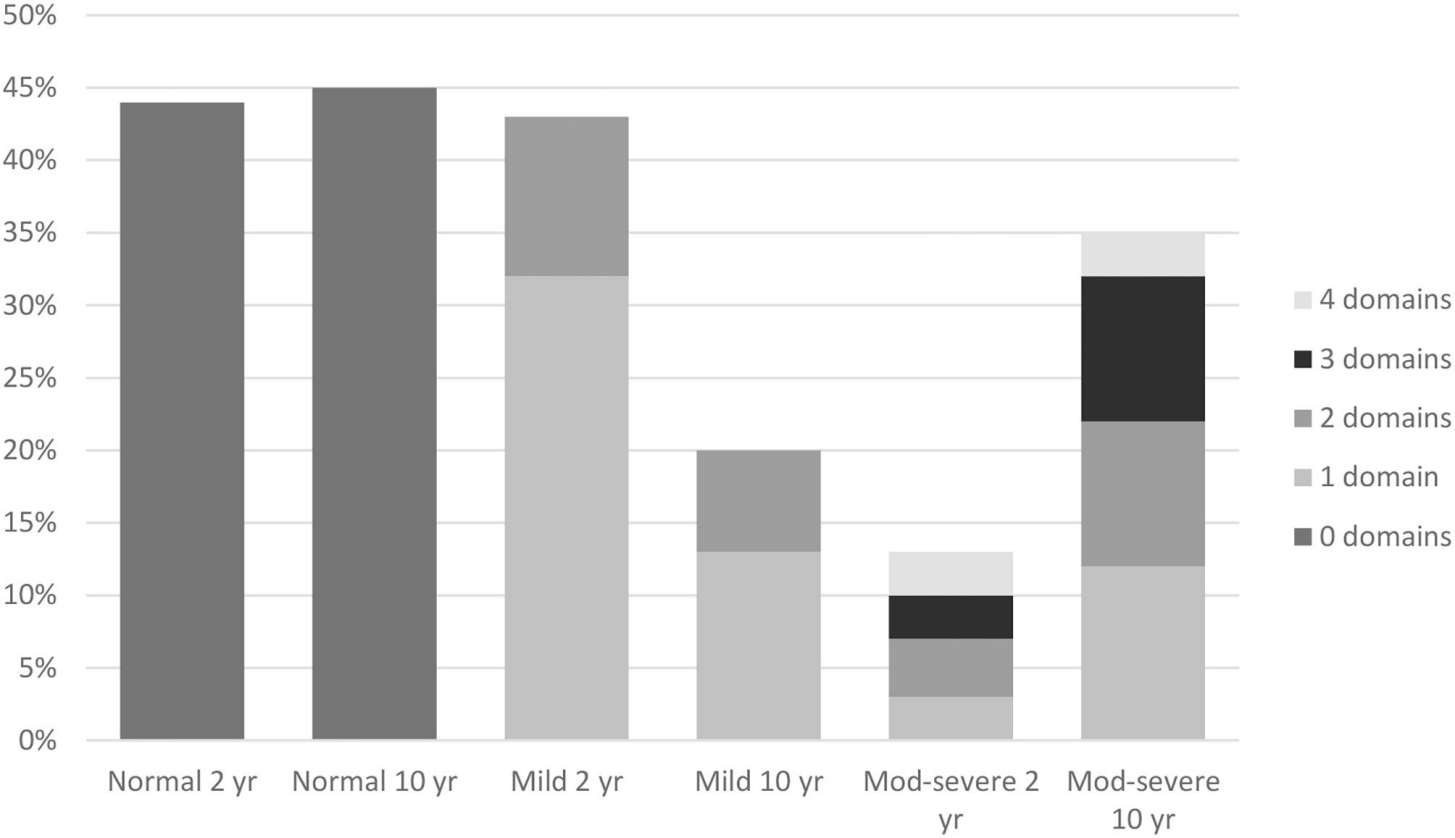
Composite impairment score and multidomain impairment score at 2 and 10 years. Normal 2 yr = no impairments at 2 years (44%). Normal 10 yr = no impairments at 10 years (45%). Mild 2 yr = at least one (32%) or multiple (11%) mild impairment(s) at 2 years. Mild 10 yr = at least one (13%) or multiple (7%) mild impairment(s) at 10 years. Mod-Severe 2 yr = at least one (3%) or multiple (10%) moderate-severe impairments at 2 years. Mod-Severe 10 yr = at least one (12%) or multiple (23%) moderate-severe impairments at 10 years.

Figure 1 shows the composite impairment score and number of affected domains (ranging from zero to four) at 2 and 10 years of age. At both time points, a comparable number of children (31 children (44%) at 2 years of age and 32 children (45%) at 10 years of age) had a normal outcome. From 2 to 10 years of age, the number of children with a mild impairment decreased, from 31 (44%) at 2 years of age to 14 (20%) at 10 years of age (*p* = 0.006). At the same time, the number of children with a moderate-severe impairment increased, from 9 (12%) at 2 years of age to 25 (35%) at 10 years of age (*p* = 0.001).

### Multidomain Impairment Score

Children with a mild impairment experienced, at both timepoints, difficulties in one or two domains, whilst children with a moderate-severe outcome experienced, at both time-points, difficulties in a range of one to all four domains (Figure 1). Although the number of children with a multidomain impairment increased from 15 (21%) at 2 years of age, to 21 (30%) at 10 years of age, this was not significant (*t* = −1.217, *p* = 0.228).

### Developmental Change

Considering the group as a whole, there were no significant changes in the distribution of normal, mild and moderate-severe development between 2 and 10 years of age within the neurological, motor, cognitive, and behavioral domain (Table 2). When assessing the individual variation for each domain, significant changes were seen in the neurological (${\text{X}}_{\left(4\right)}^{2}$ = 18.432, *p* = 0.005), motor (${\text{X}}_{\left(4\right)}^{2}$ = 27.947, *p* < 0.001), and cognitive domain (${\text{X}}_{\left(4\right)}^{2}$ = 47.781, *p* < 0.001), but not in the behavioral domain (${\text{X}}_{\left(4\right)}^{2}$ = 7.514, p = 0.111) (Tables 3a–d).

**Table 3a:** Change in the neurological outcome from 2 to 10 years of age.

**Neurological outcome at 2 years**	**Neurological outcome at 10 years**			
	**None**	**Mild**	**Moderate-severe**	**Total, *n* (%)**
None (%)	33 (75)	6 (14)^a^	5 (11)^a^	44 (63)
Mild (%)	12 (63)^b^	5 (26)	2 (11)^a^	19 (27)
Moderate-severe (%)	1 (14)^b^	2 (28)^b^	4 (58)	7 (10)
Total, *n* (%)	46 (66)	13 (19)	11 (15)	70 (100)

a *Indicates a shift toward a more severe category 13/70; 19%*.

b *Indicates a shift toward a less severe category 15/70; 21%*.

**Table 3b:** Change in motor outcome from 2 to 10 years of age.

**Motor outcome at 2 years**	**Motor outcome at 10 years**			
	**None**	**Mild**	**Moderate-severe**	**Total, *n* (%)**
None (%)	47 (92)	0	4 (8)^a^	51 (81)
Mild (%)	4 (57)^b^	1 (14)	2 (29)^a^	7 (11)
Moderate-severe (%)	0	0	5 (100)	5 (8)
Total, *n* (%)	51 (81)	1 (2)	11 (17)	63 (100)

a *Indicates a shift toward a more severe category 6/63; 10%*.

b *Indicates a shift toward a less severe category 4/63; 6%*.

**Table 3c:** Change in cognitive outcome from 2 to 10 years of age.

**Cognitive outcome at 2 years**	**Cognitive outcome at 10 years**			
	**None**	**Mild**	**Moderate-severe**	**Total, *n* (%)**
None (%)	43 (77)	11 (20)^a^	2 (3)^a^	56 (81)
Mild (%)	6 (60) ^b^	4 (40)	0	10 (15)
Moderate-severe (%)	0	0	3 (100)	3 (4)
Total, *n* (%)	49 (71)	15 (22)	5 (7)	69 (100)

*n = 70*;

a *Indicates a shift toward a more severe category 13/69; 19%*.

b *Indicates a shift toward a less severe category 6/69; 9%*.

**Table 3d:** Change in behavioral outcome from 2 to 10 years of age.

**Behavioral outcome at 2 years**	**Behavioral outcome at 10 years**			
	**None**	**Mild**	**Moderate-severe**	**Total, *n* (%)**
None (%)	44 (82)	6 (11)^a^	4 (7)^a^	54 (87)
Mild (%)	3 (60)^b^	0	2 (40)^a^	5 (8)
Moderate-severe (%)	2 (67)^b^	1 (33)^b^	0	3 (5)
Total, *n* (%)	49 (79)	7 (11)	6 (10)	62 (100)

a *Indicates a shift toward a more severe category; 12/62; 19%*.

b *Indicates a shift toward a less severe category; 6/62; 10%*.

Within the neurological domain, 40% (28/70 children) shifted in severity, of whom 46% (13/28 children) moved to a more severe category and 54% (15/28 children) to a less severe category. For the motor domain, most (84%, 53/63) children remained in the same category of severity: 16% (10/63 children) moved between categories, of whom 60% (6/10 children) from no or mild impairment at 2 years of age to a moderate-severe impairment at 10 years of age. Within the cognitive domain, 28% (19/69 children) shifted in severity. The largest change occurred in children who had a normal outcome at 2 years of age; of the 56 children who performed in a normal range at 2 years of age, 20% (11/56 children) developed a mild impairment and 3% (2/56 children) a moderate–severe impairment. Within both the motor and cognitive domain, all children with a moderate-severe impairment at 2 years of age (*n* = 5 for the motor domain, *n* = 3 for the cognitive domain) still had a moderate-severe impairment at 10 years of age. Although within the behavioral domain, the individual change of children did not differ significantly over time, parents reported higher rates of problem behavior in 22% (14/65 children) at 10 years of age, with half of them (7/14) classified as having moderate-severe behavioral problems.

## Discussion

The prospective, longitudinal design of this study provided the opportunity to investigate the rate and stability of impairment over time, within multiple domains, in individual children born very preterm. With increasing age, we found more children experiencing a moderate-severe impairment in the neurological, motor, cognitive and/or behavioral domain. On a group level, these changes in the distribution of normal, mild and moderate-severe impairment in the separate domains were not significant. However, on an individual level, there was a considerable variation in all domains, showing the relevance of long-term follow-up for preterm born children and the importance to keep track of their individual development.

In our cohort, with only 3 children (4%) born below 26 weeks' gestation and 24 (34%) below 28 weeks' gestation, we still found relatively high percentages of children with long-term impairments. A moderate-severe impairment in at least one developmental domain was present in 35% of the children at 10 years of age; an almost three-fold increase compared to 2 years of age. This shows that high rates of impairment are not limited to cohorts of children born extremely preterm, and, to fully understand the extent of the difficulties experienced by children born preterm, standardized and long-term follow-up should include outcome assessment in multiple key developmental domains.

In line with the recent study by Taylor and colleagues (11) we observed large individual shifts in outcomes between 2 and 10 years of age. However, in our cohort we did not find individual improvement of moderate-severe neurodevelopmental impairment. This could possibly be explained by differences between the examined cohorts (extreme preterm vs. very preterm), but also by the use of different tests and a different specification and classification of neurodevelopmental impairment at both timepoints. Whilst we investigated neurological functioning, motor skills, cognition and behavior at both age points, Taylor and colleagues included sensory impairments, epilepsy and (symptoms of an) autism spectrum disorder. In order to truly grasp neurodevelopment impairment in children born preterm, assess the stability between infancy and childhood, and compare the outcomes within different cohorts, a standardized classification of neurodevelopmental impairment should be developed for multiple age points. Moreover, both our study and the study by Taylor experienced a relatively high loss to follow-up, which is unfortunately not uncommon in longitudinal studies and may also have contributed to the divergent results.

Although at group level there were no significant changes in the distribution of normal, mild, and moderate-severe impairments between 2 and 10 years of age within the neurological, motor and cognitive domain, there were considerable individual changes. Most changes were seen within the neurological domain, where over one third of the children shifted, mostly from mild MND at 2 years of age to no MND at 10 years of age (12/19, 63%). The early diagnosis of mild MND therefore does not seem a reliable predictor for later neurological functioning. The assessment of motor functioning was the most stable outcome measure. All children with a moderate-severe outcome at age two still had a moderate-severe outcome at age ten, and nearly all children with a normal motor outcome at age two had a normal outcome at age ten. This is in line with the positive association between Bayley motor scores and later motor functioning in children born preterm (35). Within the cognitive domain, all children with a moderate-severe outcome at 2 years of age still had a moderate-severe outcome at 10 years of age. However, almost one fifth (19%) of the children with a normal or mild outcome at 2 years of age shifted toward a more severe category over time. For the behavioral domain, none of the children with moderate-severe behavioral problems at age two had a moderate-severe behavioral outcome at age ten. However, the number of children with reported behavioral problems at age 10 remained high (21%) and those classified with moderate-severe behavioral problems all had a confirmed psychiatric diagnosis or were under assessment for a possible psychiatric diagnosis. Problem behavior in children born preterm is negatively associated with academic performance, work outcomes and family formation later in life (36, 37). The individual changes seen between 2 and 10 years of age support the importance of including a behavioral assessment at a later age to accurately identify those who have adjustment problems in later life.

### Strengths and Limitations

Strengths of the current study are the longitudinal design, enabling us to compare early outcomes with school-age outcomes within the same cohort as well as in the individual child, the inclusion of a complete and standardized neurological examination, and the inclusion of behavioral assessment, providing a better understanding of the overall occurrence of impairments in children born preterm. Knowledge on the changes in individual development in multiple domains is important for several reasons. It can help to inform the parents of children who were born preterm about the risk of persisting problems in later life. It may also help to identify those at risk and who may benefit from early intervention. Furthermore, the awareness that individual changes are common and that outcomes at a young age may not reflect outcomes in later life is important when investigating the associations between neonatal risk factors and neonatal treatment strategies and neurodevelopmental outcome.

A limitation of our study is the lack of a healthy term-born control group in order to control for change in outcome in children born full-term. However, we used standardized assessments and compared our study population with age-appropriate normative means. The different assessment tools available for children aged two and ten, may have played a role in the variation in measured outcome. Still, the assessments used in this study reflect current clinical practice at both timepoints. Due to the original design of the study (investigating brain imaging findings in a prospective cohort of children born very preterm), no sample size or power analysis was performed for loss to follow-up at 2 and 10 years of age. This resulted in a relatively small sample size. Possibly due to the large time-interval between both assessments only 71 children of the original cohort could be assessed at both timepoints. Since previous studies reported rates up to 75% of preterm born children experiencing one impairment and a co-occurrence of impairments in up to 52% at preschool age (38–40), and others already suggested weak agreement between neurodevelopmental disabilities in infancy and early childhood (6, 7), future studies should also include follow-up assessment around 5 years of age. This will provide more information on what happens between toddlerhood, early childhood and school-age and on the critical periods of development in different domains in children. This may give more information on targeted age-points for intervention. Moreover, due to the large time-interval between both assessments and the variance in outcomes of individual children between the two time-points, we did not use multiple imputation as this may lead to an under- or over estimation of outcomes. In longitudinal cohorts with a shorter time-interval between assessments and more follow-up timepoints, multiple imputation can be used to correct for missing data. Finally, as the assessments were part of the clinical follow-up program, the assessors were not blinded to important perinatal details of the participants such as gestational age at birth and birthweight.

In conclusion, our results indicate that long-term follow-up in a broad range of developmental domains in children born very preterm is clinically relevant and should be continued up to at least school-age, and possibly also into adolescence. Children with a moderate-severe impairment at 2 years of age in the motor and/or cognitive domain are likely experiencing moderate-severe impairments within the same domain at 10 years of age. However, normal and mild abnormal outcomes at 2 years of age should be interpreted with care, since there are large individual shifts over time. As these results are a less reliable predictor for development at school age these children should therefore not be discharged from follow-up care too soon.

## Data Availability Statement

The raw data supporting the conclusions of this article will be made available by the authors upon reasonable request.

## Ethics Statement

The studies involving human participants were reviewed and approved by Medical ethics committee (CME) of the Leiden University Medical Center. Written informed consent for participation was not provided by the participants' legal guardians/next of kin because: The institutional review board of the LUMC approved this study, and written parental consent was obtained from both parents at term age and for follow-up 2 years of age (P06.002). For the follow-up at 10 years of age, a waiver was obtained as this was part of the national clinical follow-up program (C15.072/P17.087).

## Author Contributions

Data collection and analysis were performed by LJ, CP-S, MR, SS, and JE-vD. AB-H was involved in the methodological design of the study and also contributed to the statistical analysis of the manuscript. The first draft of the manuscript was written by LJ. Supervision was provided by JK, CP-S, RV, and SS. JK contributed to the data collection. All authors commented on previous versions of the manuscript, contributed to the study conception and design, and read and approved the final manuscript.

## Conflict of Interest

The authors declare that this study received funding from Chiesi Pharmaceutical Ltd. The funder was not involved in the study design, collection, analysis, interpretation of data, the writing of this article or the decision to submit it for publication.

## Abbreviations

BPD: bronchopulmonary dysplasia
BW: birth weight
BSID: Bayley scale of infant and toddler development
CP: cerebral palsy
CBCL: child behavior checklist
GA: gestational age
GMFCS: gross motor function classification score
IVH: intraventricular hemorrhage
M-ABC: movement assessment battery for children
MND: minor neurological dysfunction
NEC: necrotizing enterocolitis
SD: standard deviation
SGA: small for gestational age
WISC: Wechsler intelligence scale for children.
